# 

**Published:** 1895-05

**Authors:** 


### SELECTIONS.

Article I.—The Dentist: His Relations to His Patients and to His Brother Practitioners. By Dr. C. S. Lane. - 1

" II.—Dentistry and General Medicine. By George H. Chance, D. D. S. - - - - - - - 5

" III.—The Horace Wells Permanent Memorial. - - 9

" IV.—Preventive Dentistry. By I. P. Wilson, D. D. S. - 10

" V.—A Few More Words on Pyorrhœa. By Dr. A. V. Elliott. - - - - - - - - 13

" VI.—Oxyphosphate of Zinc as a Filling Material.—Its Abuse. By J. E. Wilkinson, D. D. S. - - 21

" VII.—Extracts from Essays on Chinese Dentistry. By A. M. H. through C. Robbing, L. D. S. - - 24

" VIII.—The Preparation of Roots for Crowning and Gold Crowns. By Dr Campbell. - - - - - 29

" IX.—The Real Value of the Medicinal Peroxide of Hodrogen Preparations Found in the Market. By H. Endemann, Ph. D. - - - - - - 35

" X.—A Case in Practice. By Prof. W. D. Miller, M. D., D. D. S. - - - - - - - - - - 37

" XI.—Medical Men and Dentistry. By Dr. Geo. W. Warren. - - - - - - - - 38

### EDITORIAL, ETC.

Dental Surgery. - - - - - - - - - - - - 40

Program of the Second Union Meeting of the Washington City Dental Society and the Maryland State Dental Association, at the Royal Arcanum Hall, Baltimore, Md. - - - - 41

### MONTHLY SUMMARY.

"Dental Medicine." - - - - - - - - 45

A Note on Capping a Tooth Pulp. - - - - - - 46

Copper Amalgam. - - - - - - - - - - - 46

The Necessity of a Higher Estimation of Tooth and Mouth Hygiene. 47

Vitality Conditioned. - - - - - - - - - - 47

Some Queer Questions Regarding the Teeth. - - - - - 48

Dr. Cunningham on Tooth Powders. - - - - - - 48

